# A Chemoinformatics Approach to the Discovery of Lead-Like Molecules from Marine and Microbial Sources *En Route* to Antitumor and Antibiotic Drugs

**DOI:** 10.3390/md12020757

**Published:** 2014-01-27

**Authors:** Florbela Pereira, Diogo A. R. S. Latino, Susana P. Gaudêncio

**Affiliations:** 1 CQFB (Centro de Química Fina e Biotecnologia)/REQUIMTE, Departamento de Química, Faculdade de Ciências e Tecnologia, Universidade Nova de Lisboa Campus Caparica, Caparica 2829-516, Portugal; E-Mails: dalatino@fc.ul.pt (D.A.R.S.L.); s.gaudencio@fct.unl.pt (S.P.G.); 2 CCMM (Centro de Ciências Moleculares e Materiais), Departamento de Química e Bioquímica, Faculdade de Ciências, Universida Lisboa, Campo Grande, Lisboa 1749-016, Portugal

**Keywords:** quantitative structure–activity relationships (QSAR), marine natural products, microbial natural products, antibiotic, antitumor, drug discovery

## Abstract

The comprehensive information of small molecules and their biological activities in the PubChem database allows chemoinformatic researchers to access and make use of large-scale biological activity data to improve the precision of drug profiling. A Quantitative Structure–Activity Relationship approach, for classification, was used for the prediction of active/inactive compounds relatively to overall biological activity, antitumor and antibiotic activities using a data set of 1804 compounds from PubChem. Using the best classification models for antibiotic and antitumor activities a data set of marine and microbial natural products from the AntiMarin database were screened—57 and 16 new lead compounds for antibiotic and antitumor drug design were proposed, respectively. All compounds proposed by our approach are classified as non-antibiotic and non-antitumor compounds in the AntiMarin database. Recently several of the lead-like compounds proposed by us were reported as being active in the literature.

## 1. Introduction

Small organic molecules have proven to be important tools for biological systems research, but there is still much to learn from their use. From the 150,000 structures in the CRC Dictionary of Natural Products (NPs) only approximately 1% of them have at least one biological test results described in the MDL Drug Data Report database (MDDR) [1], which has been compiled by Prous Science from the patent literature since 1988. NPs or synthetic products inspired by NPs have been the single most productive source leads for the development of drugs. In fact, more than half of the approved drugs from 1981 to 2010 are based on NPs [2]. At the turn of the 21st century, a new branch of NPs chemistry had been fully established—Marine Natural Products (MNPs). In fact, the future seems very promising for this new branch since MNP chemists have already elucidated the chemical structure of over 22,000 novel compounds [3]. Moreover, from these, 7 are already approved drugs [4], (four anticancer, one antiviral, one pain control, and one hypertriglyceridemia). The success rate of drug discovery from the marine world is 1 drug per 3140 natural products described. This rate is approximately 1.7- to 3.3-fold better than the industry average (1 in 5000–10,000 tested compounds) [5]. Nowadays, there are facilities for high-throughput screening available both in academic labs or in drug pharmaceutical companies, but the cost of random screening for very large collections of compounds can nevertheless be prohibitive, making chemoinformatics approaches for the virtual screening of the most probable active compounds valuable tools. Although in the last few years chemoinformatics approaches have been applied for *in silico* screening of natural products [6,7,8,9,10,11,12], this is a field that can be significantly improved by the modeling of data from large databases containing information relative to biological activities, which are becoming available to the scientific community. Besides the virtual screening, the computational methods can be very useful to build appropriate databases, allowing for effective short-circuits of NP extracts dereplication procedures [13], which will certainly result in increasing the efficiency of drug discovery. An example of a powerful database is AntiMarin [14], which contains approximately 50,000 compounds from marine macroorganisms and both marine and terrestrial microorganisms. AntiMarin is the result of a merger between AntiBase [15] (a database of all terrestrial and marine microbial natural products) and MarinLit [3] (a database of marine natural products literature). Bacteria are an exceptional source of chemical diversity. Among them, species of the order *Actinomycetales* (commonly called actinomycetes) are the single most productive source of microbial derived natural products, accounting for *ca.* 75% of all antibiotics discovered [16], as well as a broad range of anticancer agents. Although the actinomycetes are best known as soil bacteria, a growing interest in its distribution and ecological role in the marine environment has been observed. Therefore, besides marine natural products (MNPs) we will refer the NP derived from microbial source as microbial natural products M_b_NPs, accounting for those of terrestrial origin.

In this work, we explored the application of machine learning (ML) techniques to exploit lead-like molecules *en route* to antitumor and antibiotic drugs from 1192 MNPs and M_b_NPs (extracted from the AntiMarin database). The models were developed using 1804 active and non-active compounds from the PubChem database. Support Vector Machines (SVMs), Random Forests (RFs) and Classification Tree (CTs) models, were compared to predict the two classes (*i.e.*, active and non-active compounds) in the following classification task: (1) the overall biological activity; (2) the antitumor activity; and (3) the antibiotic activities from CDK descriptors.

## 2. Results and Discussion

### 2.1. Establishment of Quantitative Structure–Activity Relationship (QSAR) Classification Models

The results for internal validation (ten-fold cross-validation with SVM and out-of-bag estimation with RF on the training set) and external validation (on test set I) are presented in Table 1. A further test set (test set II) extracted from the AntiMarin database was also used but not with the main purpose of external model validation. The AntiMarin data set was screened by the developed models to find lead-like molecules *en route* to antitumor and antibiotic drugs, which, indeed, is the core of the presented work. RFs showed a better performance when compared to a single CT and SVMs in the prediction of the overall biological, antitumor, and antibiotic activities for test set I, taking into account the value of the G-mean (Table 1).

**Table 1:** Comparison of different machine learning techniques for building QSAR classification models with CDK descriptors.

ML		SVM ^a^	RF ^b^	CT
**Training set/Test Set I/Test Set II**				
**Model**	**Class Size**	**Correct Predictions Sensitivity** **^c^** **Specificity** **^d^** **G-mean** **^e^**	**Correct Predictions Sensitivity** **^c^** **Specificity** **^d^** **G-mean** **^e^**	**Correct Predictions Sensitivity** **^c^** **Specificity** **^d^** **G-mean** **^e^**
Overall	YES ^f^ 1666/834/879 NO ^g^ 138/68/313	YES 1295/647/671 NO 73/37/80 0.78/0.78/0.76 0.53/0.54/0.26 0.64/0.65/0.44	YES 1152/591/660 NO 87/53/101 0.69/0.71/0.75 0.63/0.78/0.32 0.66/0.74/0.49	YES 1657/821/864 NO 24/3/3 0.99/0.98/0.98 0.17/0.04/0.01 0.42/0.21/0.10
Antitumor	YES 918/456/208 NO 886/446/984	YES 744/377/79 NO 648/337/650 0.81/0.83/0.38 0.73/0.76/0.66 0.77/0.79/0.50	YES 792/388/86 NO 689/357/686 0.86/0.85/0.41 0.78/0.80/0.70 0.82/0.82/0.54	YES 775/369/84 NO 617/295/643 0.84/0.81/0.40 0.70/0.66/0.65 0.77/0.73/0.51
Antibiotic	YES 654/338/625 NO 1150/564/567	YES 501/262/419 NO 1061/516/337 0.77/0.78/0.67 0.92/0.91/0.59 0.84/0.84/0.63	YES 548/271/433 NO 1090/531/376 0.84/0.80/0.69 0.95/0.94/0.66 0.89/0.87/0.68	YES 521/249/391 NO 1094/516/396 0.80/0.74/0.63 0.95/0.91/0.70 0.87/0.82/0.66

^a^ Ten-fold cross-validation; ^b^ out-of-bag; ^c^ the ratio of true positives to the sum of true positives and false negatives; ^d^ the ratio of true negatives to the sum of true negatives and false positives; ^e^ the square root of the product of sensitivity and specificity; ^f^ active; ^g^ non-active.

As expected the predictive power of CT is lower than that obtained for the other two ML techniques for the three activity models, but has the advantage in establishing a few simple rules that provide insights into the drug profile.

#### 2.1.1. Overall Biological Activity Model

**Figure 1:**
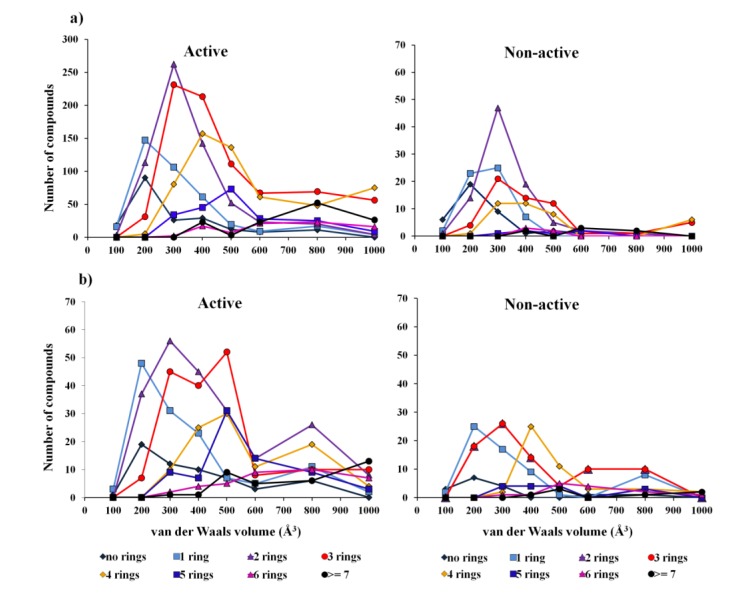
Comparison of the van der Waals volume of: (**a**) 2706 PubChem compounds, and (**b**) 1192 AntiMarin compounds containing different ring numbers.

For the overall biological activity model the data are imbalanced as concerns the active and non-active classes, and therefore represents a problem for the CT method, which is not able to balance the two classes, as has been done with the other two methods (*i.e.*, by adjusting the weight of each class). As was expected the overall CT biological model shows poor prediction accuracy—a G-mean of 0.21 for test set I. With the overall biological activity model, we intended to improve the precision of lead-like profiling by compensating for the lack of bioactivity records in the 1192 compounds extracted from the AntiMarin database. Like in other databases, in the AntiMarin database each molecule has been tested for only a few activities chosen by the researchers or grant programs, so there are probably many bioactive molecules that lack an activity record, because they have never been tested against other biological screens. With this problem in mind we would expect that the difficulty with this model was actually the false positives (FPs) for the test set II. Indeed, we obtained a large number of FP for the test set II, as shown by the low specificity value of 0.32 obtained for the best model (RF) compared with the high specificity value of 0.78 obtained for the test set I, Table 1. The analysis of the results obtained from RF and SVM models has shown that there are 176 and 12 FP, common to both models, for the test set II and I, respectively. For these FP, it was obtained an average probability of being active (Prob_ave_act) in the RF model of 0.68 (80 FP have a Prob_ave_act ≥ 0.7), and 0.61 (2 FP have a Prob_ave_act ≥ 0.7) for the test set II and I, respectively. Many authors have tried to rationalize the drug-like and lead-like nature of compounds, Waldmann *et al.* [8] suggested through a statistical analysis of the structural classification of natural products that more than half of all natural products have just the right size to serve as a starting point for hit and lead discovery. The lead-like molecules must have a scaffold with two, three, or four rings, and their van der Waals volumes must match the lower end of the majority of the protein cavities (*i.e.*, van der Waals volume between 300 and 800 Å^3^ [8]). The analysis of the active and non-active profiles of the van der Waals volume of the 2706 compounds from PubChem (training set and test set I) and 1192 compounds from AntiMarin (test set II) containing different ring numbers demonstrated that the non-active compounds from AntiMarin present a higher number of compounds having three and four rings with the right size than those from PubChem (Figure 1).

In fact, it appears that there is a correlation between active compounds and 3-, 4-ringed compounds with a van der Waals volume between 300 and 800 Å^3^. In the test set I, there are 225 compounds with these specifications. From those 216 compounds (*i.e.*, approximately 96%) are active compared with only 195 active compounds (*i.e.*, approximately 72%) from the 271 AntiMarin (test set II) compounds with the same specifications. On the other hand 188 and 178 compounds were predicted as true positive (TP) with a Prob_ave_act of 0.72 and 0.74 using the RF model for test set I and II, respectively. Moreover, for the test set II, 67 compounds were predicted as FPs, compared with only 4 FPs for the test set I, with a Prob_ave_act of 0.68 and 0.59, respectively. Additionally, we use the Lipinski rule-of-five (R-o-5) to analyze the RF activity model. The R-o-5 is widely used as a filter in drug discovery to evaluate drug likeness and to verify if a compound with some activity has properties that would make it a possible orally active drug in humans. It is applied in the optimization of activity, selectivity and drug-like properties of lead compounds. Beside its wide application, it should be emphasized that the R-o-5 does not really predict if a compound is active, the Lipinski rule states that a compound that is more likely to be an orally active drug and easily absorbed by the body has no more than one violation of the following criteria: a molecular weight less than 500, an estimation of the octanol-water partition coefficient not greater than 5, a sum of nitrogen and oxygen atoms in a molecule not more than 10 and, a total number of potential hydrogen-bond donors not more than 5 [17]. This criterion was applied to test set II and the filtered compounds that conform to the R-o-5 were analyzed and compared to the RF performance for the same set of filtered compounds. Similarly to the RF model with the application of the R-o-5 to test set II a large number of FPs emerged, 202 (212 FP by the RF model). From those Lipinski’s FP compounds there were as well 108 compounds that were predicted as FP by the RF model with a Prob_ave_act of 0.61.

**Scheme 1:**
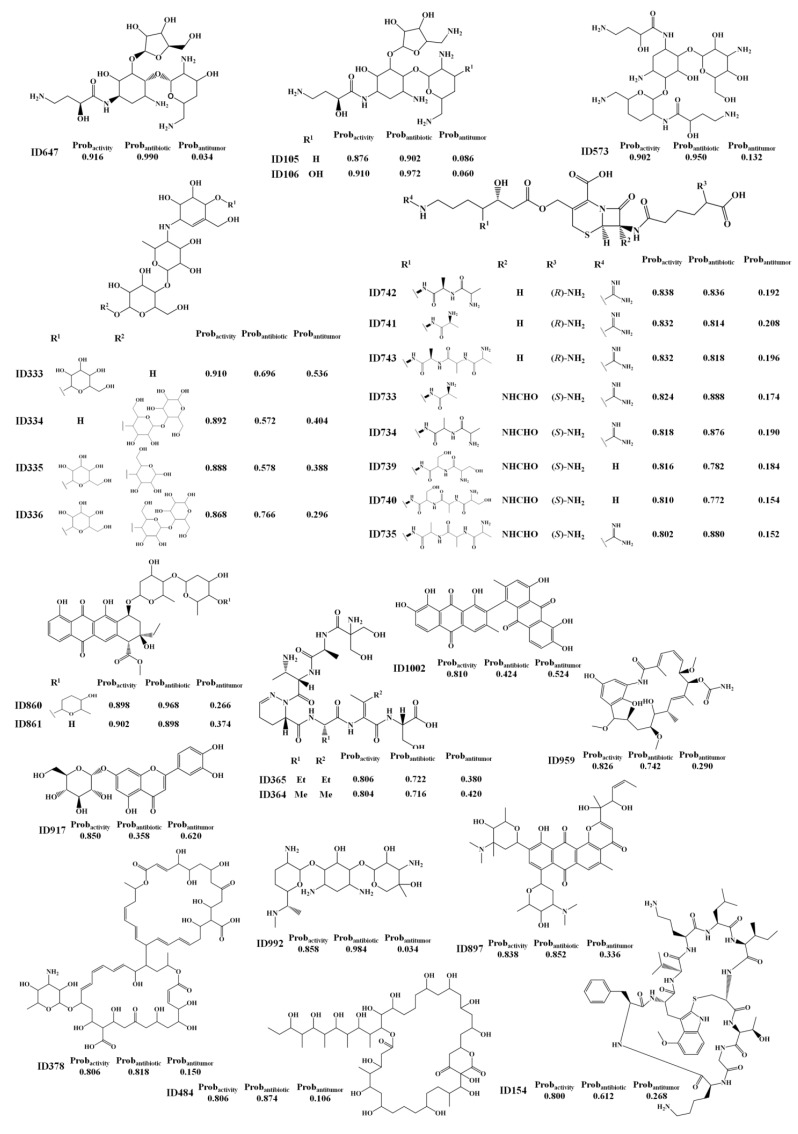
The selected 28 lead bioactive MNPs and M_b_NPs from the AntiMarin database using theRF activity model.

Twenty eight MNPs (Scheme 1) were listed as having a probability of being active (Prob_activity_) greater than or equal to 0.8 using the RF activity model. For the test set I, only active compounds were predicted with a Prob_activity_ greater than or equal to 0.8 using the RF activity model. We propose these MNPs and M_b_NPs as lead bioactive compounds and consider that they have been misclassified as non-active compounds in the AntiMarin database.

From these 28 FPs, seven compounds (IDs 105, 106; 333, 573, 647, 917 and 992) are considered to be FPs using the “right size” approach but only one (ID 959) is conform to the R-o-5 criteria. Interestingly, from the 28 FPs, 26 MNPs and M_b_NPs (*i.e.*, 25 M_b_NPs and 1 MNP) were classified as being antibiotic by the RF antibiotic model with an average probability of being antibiotic (Prob_ave_antibiotic) of 0.82, compared with only 3 MNPs and M_b_NPs (*i.e.*, 1 M_b_NP and 2 MNPs) that were classified as being antitumor by the RF antitumor model with an average probability of being antitumor (Prob_ave_antitumor) of 0.56. From those 26 FPs, 6 (IDs 105, 106, 573, 647, 860 and 992) were predicted as being antibiotic with a probability of being antibiotic (Prob_antibiotic_) greater than or equal to 0.9 using the RF antibiotic model. Although no biological activity was recorded for the butirosin A analogs (IDs 105, 106 and 647, see Scheme 1) in the AntiMarin database, the work of Woo *et al.* [18] had shown an improved antibiotic activity of deoxy derivatives in the α-d-glucopyrasonyl unit (namely IDs 105, 106 and 647) over butirosin A (a broad-spectrum aminoglycoside antibiotic). Moreover, this study had referred also to the advantageous effect of the 5-amino function in the β-d-xylofuranosyl unit (e.g., IDs 105, 106) as compared with the 5-hydroxyl group of butirosin A and M_b_NP ID 647, against some, but not all, of the organisms studied. The 1-*N*-acyl derivative of the arbekacin (ID 573 see Scheme 1), was identified as being resistant to many inactivating enzymes while retaining most of the intrinsic antibiotic activity of the unsubstituted molecules against susceptible strains [19]. As far as we know, the bioactivity of the 3′-*N*-demethylated derivative (ID 992, see Scheme 1) of gentamicin C1 (a naturally occurring broad-spectrum antibiotic) has never been recorded. However in accordance with the literature we expect that this derivative may be active since it carries a methyl group on *N*-6′, which appears to be responsible for the resistance to *N*-acetyltransferase enzymes as referred for the gentamicin C1 and C2b [19]. The aklavinone glycoside, ID 860 Scheme 1, is a parent aglycone of an extensive family of glycosidically derived anthracycline antibiotics possessing significant anticancer activity, although the specific activity of this M_b_NP has not yet been reported. The aglycone, luteolin, of the flavonoid, luteolin-7-*O*-β-glucoside (ID 917, Scheme 1) has been shown to inhibit a series of human cancer cell lines [20,21] (renal A-549, ovary SK-OV-3, melanoma SK-MEL-2, XF-498, HCT15, gastric HGC-27), breast MCF-7 [22] and human leukemia cells [23]. More recently, additional antitumor assays have suggested that luteolin-7-*O*-β-glucoside could be an interesting antitumor NP, even more active in the breast adenocarcinoma cell than its aglycone [24]. Although the bioactivity of the MNP, ID154, has never been recorded, the high toxicity of those cortinarin derivatives was extensively reported [25]. 

#### 2.1.2. Antitumor Activity Model

**Scheme 2:**
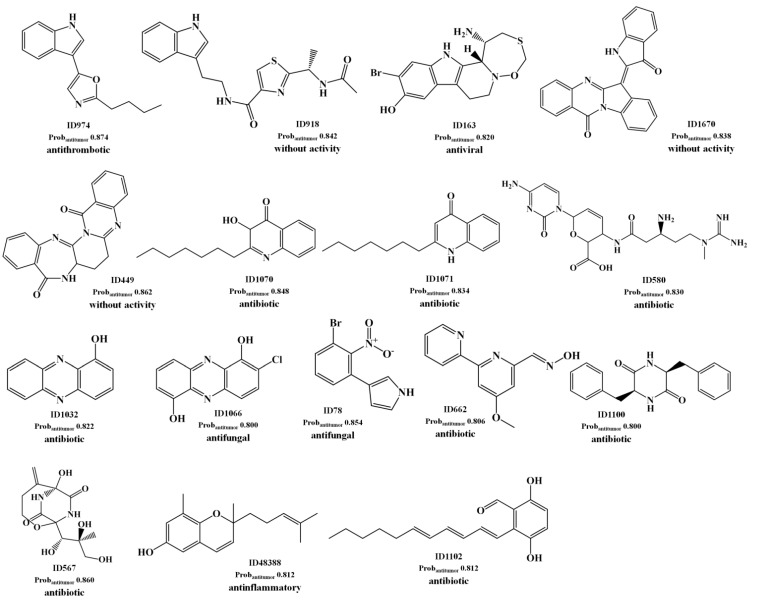
The selected 16 lead antitumor MNPs and M_b_NPs from the AntiMarin database using the RF antitumor model.

For the best antitumor model (RF model) we obtained a large number of FPs as well as FNs for the test set II, as shown by the low sensitivity and specificity values of 0.41 and 0.70, respectively. These values could be compared with the sensitivity and specificity values of 0.85 and 0.80 obtained for the test set I, respectively Table 1. The analysis of the predictions obtained for the test set II has shown that there are 86 TP with a Prob_ave_antitumor of 0.62, as compared with a Prob_ave_antitumor of 0.63 obtained for the 298 FPs predicted. In our opinion, these FPs are not all misclassifications by the antitumor model. Instead those with high probability of being antitumor (Prob_antitumor_) are lead-like antitumor MNPs and M_b_NPs, which have been misclassified as non-antitumor compounds in the AntiMarin database. These conclusions were supported by the low percentage of misclassifications as FP (4.90%) obtained for the test set I compounds with a Prob_antitumor_ greater than or equal to 0.8 using the RF antitumor model. In the Scheme 2 were listed the 16 FPs with a Prob_antitumor_ greater than or equal to 0.8 using the RF antitumor model.

We proposed these MNPs as lead antitumor compounds although no antitumor activity was recorded in the AntiMarin database. From these 16 FP, 8 are MNPs and the others are M_b_NPs. However, antitumor activity was reported more recently, for almost half of the 16 FP that we obtained, namely (see Scheme 2): the benzodiazepine derivative (ID 449) against NCI-H292 human lung carcinoma cells [26], the indole alkaloid ID 918 against K-562 human leukemia and HeLa human cervix carcinoma cells [27], the phenazine derivatives ID 1032 as a light-activated tumor cytotoxic compound [28] and ID 1066 against LXFL 529L human lung carcinoma cells [29], the oxathiazepine derivative ID 163 as potent antitumor [30], the polyketide metabolite ID 1102 against NCI-H187 human lung cancer cells [31], and the oxime derivative ID 662 against several NCI human tumor cells [32].

**Scheme 3:**
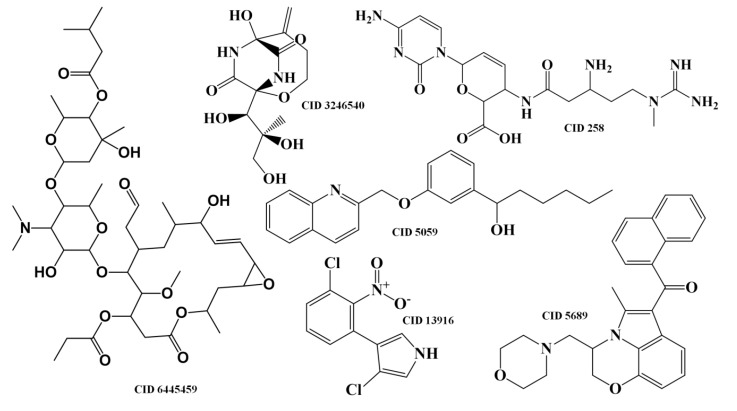
The reported compounds from training and test sets.

Taking into account the measure of similarity by the RF antitumor model between the 1804 compounds of the training set and the 1192 compounds of the test set II—the maximum proximity (Prox_max_) values between training and test set II compounds—it was possible to establish the following correlations: the antithrombotic indole alkaloid ID 974 (Scheme 2) presents a Prox_max_ of 0.786 with the antithrombotic and antitumor α-pentyl-3-(2-quinolylmethoxy)-benzenemethanol, CID 5059 from the training set (see Scheme 3); the antibiotic diketopiperazine ID 567 (Scheme 2) presents a Prox_max_ of 0.888 with one of its stereoisomers that was reported to be antibiotic and antitumor, CID 3246540 from the training set (Scheme 3); the 3-*S* stereoisomer ID 580 (Scheme 2) presents a Prox_max_ of 0.916 with its racemate, CID 258 from the training set (Scheme 3), which was reported to be antitumor. The antifungal brominated phenylpyrrole derivative, ID 78 (Scheme 2), is related to the chlorinated pyrrolnitrin, an antibiotic and antifungal MNP from test set I (CID 13916, see Scheme 3) that was recently reported as active in screenings for potential anti-tumor activity [33]. Moreover both phenylpyrrole derivatives (*i.e.*, ID 78 from test set II and CID 13916 from test set I) were predicted as being antitumor by the RF model with a Prob_antitumor_ of 0.85 and 0.83, respectively. The anti-inflammatory ID 48388 (Scheme 2) MNP is a potent inhibitor in both enzymatic assays 5-lipoxygenase and cyclooxygenase-1 [34].

From its chemical structure (similar to vitamin E), it is probable that it helps to protect against the damaging effects of free radicals, which are well known to possibly contribute to the development of chronic diseases such as cancer [35]. Despite that the tryptanthrin analog ID 670 (Scheme 2) has been reported as not possessing *in vivo* antitumor activity against human tumor cell lines (MCF-7 breast, NCI-H460 lung, SF-268 central nervous system) [36], it was predicted to be antitumor by the RF model with a Prob_antitumor_ of 0.84. In this model, the MNP ID 670 presents a Prox_max_ of 0.44 with the analgesic and antitumor cannabinoid, CID 5689 from the training set (Scheme 3). The cannabinoid, CID 5689 has been reported to possess antimitogenic activity on human melanoma cells (COLO38 and OCM-1) [37]. With respect to FN, the analysis of the predictions obtained for the test set II has shown that there are 686 TNs with a Prob_ave_antitumor of 0.26, compared with a Prob_ave_antitumor of 0.31 obtained for the 122 FNs predicted. From these 122 FN only 10 M_b_NPs were predicted with a Prob_antitumor_ lower than 0.15. Interestingly 6 of these compounds (IDs 863, 354, 905, 444, 273 and 684) belong to the class of anthracycline antibiotics, one of the most widely used classes of antitumor antibiotics. Those 6 FNs are isolated from the actinomycete bacteria of the genus *Streptomyces*.

#### 2.1.3. Antibiotic Activity Model

**Scheme 4:**
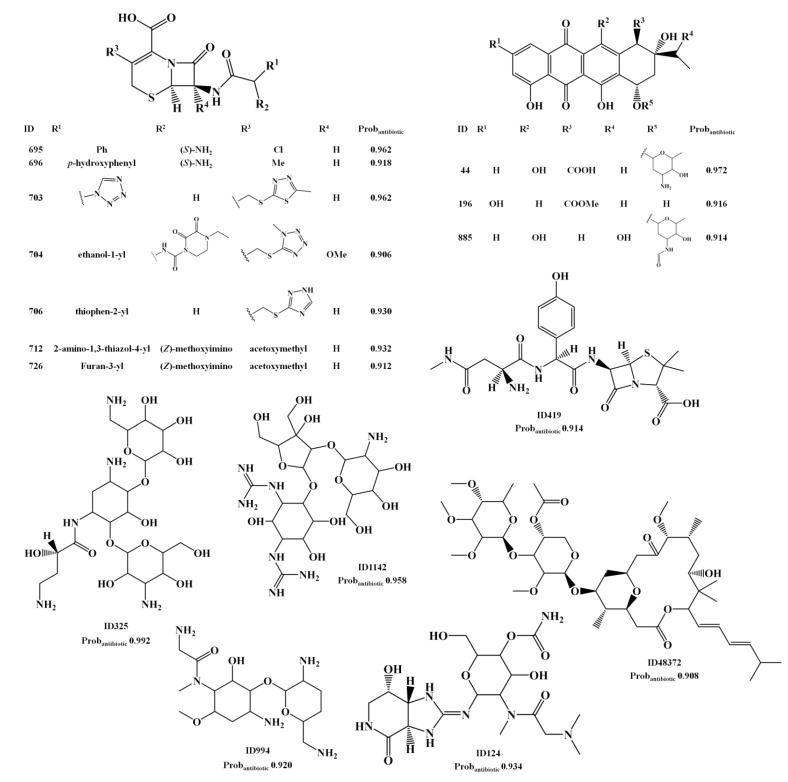
The unreported 18 lead antibiotic MNPs and M_b_NPs from AntiMarin database using the RF antitumor model with a Prob_antibiotic_ greater than or equal to 0.9.

The best antibiotic model (RF model) yielded a large number of FPs as well as FNs for the test set II, as shown by the low sensitivity and specificity values of 0.69 and 0.66 obtained, respectively. These values could be compared with the sensitivity and specificity values of 0.80 and 0.94 obtained, respectively, for the test set I, Table 1. The analysis of the predictions obtained for the test set II has shown that there are 433 TPs with a Prob_ave_antibiotic of 0.80, as compared with a Prob_ave_antibiotic of 0.71 obtained for the 191 FP predicted. In our opinion, these FP are not all misclassifications by the antibiotic model but instead those with high Prob_antibiotic_ may be lead-like antibiotic MNP and M_b_NP, which have been misclassified as non-antibiotic compounds in the AntiMarin database. For the test set I, 96.62% of compounds with a Prob_antibiotic_ greater than or equal to 0.8 were predicted as TP by the RF model. As we had done for the antitumor and biological overall models, we proposed 57 FPs with a Prob_antibiotic_ greater than or equal to 0.8 for the test set II as lead antibiotic compounds in Table S1, given in Supplementary Information. The analysis of the predictions obtained for test set II has shown that there are 23 FP with a Prob_antibiotic_ greater than or equal to 0.9. From those 23 FPs, 6 FPs (IDs 105, 106, 573, 647, 860 and 992) have been already analyzed in the overall biological model (Scheme 1). Scheme 4 illustrates the 18 FP with a Prob_antibiotic_ greater than or equal to 0.9, which have not yet been reported in this work.

Several cephalosporin analogs that appear in the AntiMarin database without activity records, were classified as actives by the overall activity and antibiotic models indicating that the cephalosporin core structure appears to be relevant to the antibiotic activity. The structures could be seen in Scheme 1 (IDs 733, 734, 735, 739, 740, 741, 742 and 743) and Scheme 4 (IDs 695, 696, 703, 704, 706, 712 and 726). However, it is not a surprising outcome, since penicillin and cephalosporin are well known antibiotic drugs and the earliest antibiotics discovered. A penicillin analog was also classified as FP, ID 419 in Scheme 4. Among other relevant types of antibiotics that were classified as FP in both models are the aminoglycosides (IDs 325, 994) and the anthracycline antibiotics (IDs 44, 196 and 885). Three guanidinyl derivatives (IDs 124, 327 and 1142) were also classified as being antibiotic. Although the lack of reported antibiotic activity for 2-aminopurine derivative, ID 327 (Scheme 4), it has been suggested, taking in account its remarkable activity against *Pyricularia oryzae* (a causative agent for rice blast disease) and the fact that several antibiotics (e.g., blasticidins S, kasugmycin, bramycin, miharamycin, aabomycin A) possess the same activity, that 2-aminopurine derivative (ID 327) may be a likely candidate [38]. 

The herbicidal and antimicrobial compound ID 124 (Scheme 4) was reported to possess lower activity than its *N*-methylglycine analog, ID 142 [39]. ID 142 and ID 124 were predicted as being antibiotic, by the RF antibiotic model, with probabilities of being antibiotics of 0.96 and 0.94, respectively. Weak antibacterial activity was reported for the guanidinylated aminoglycoside ID 1142 [40] although it appears in the AntiMarin database only with antimicrobial activity. The polycarvenoside A, analog of the macrolide ID 48373 (see Scheme 4), is a human lethal toxin [41]. Finally the MNP, ID 48373, and the maridomycin I, CID 6445459 (see Scheme 3), present similar structures and a proximity value of 0.74.

### 2.2. Analysis of Molecular Descriptors Identified as Relevant for Modeling Overall Biological Activity, Antitumor and Antibiotic Activities

The sets of descriptors selected by the CFS filter to develop the models were presented in Table 2. All models were built using CPSA (charge partial surface area) [42], topological, constitutional and molecular descriptors. The TopoPSA—topological polar surface area—descriptor [43] was selected to be used in all models. The ten most relevant descriptors, found by the RF algorithm, used to build the RF models also include in almost cases the different types of descriptors mentioned above.

**Table 2:** The comparison of descriptor selected with descriptor importance using to build QSAR models for the prediction of overall activity, antitumor and antibiotic activities.

Model			CDK Descriptors
Overall biological activity	SVM ^a^		20D: ALogp2; BCUTc-1l; BCUTp-1h; PPSA-2; FPSA-3; TPSA; RHSA; Wlambda2.unity; ATSc3; ATSc4; C3SP2; SCH-5; SP-6; VP-7; khs.ssCH2; khs.dsCH; khs.sssCH; khs.sNH2; MDEC-33; TopoPSA
	RF	MeanDecreaseAccuracy ^b^	Weta1.unity; FMF; BCUTw-1l; HybRatio; PNSA-2; ATSm1; FPSA-1; bpol; BCUTp-1l; TPSA
		MeanDecreaseGini ^b^	Weta1.unity; TopoPSA; TPSA; FNSA-2; ALogp2; LOBMAX; FPSA-2; ATSc3; PNSA-2; Weta3.unity
	CT		7D: SP-6; BCUTc-1h; Wnu1.unity; Weta1.unity; SC-5; ATSc4; PPSA-3
Antitumor activity	SVM ^a^		43D: ALogp2; AMR; BCUTw-1h; BCUTp-1l; PNSA-3; FPSA-3; FNSA-2; WNSA-3; TPSA; naAromAtom; nAromBond; ATSc2; ATSc3; ATSc4; ATSc5; ATSm5; bpol; C1SP2; C2SP2; SCH-4; VCH-4; VCH-5; VCH-7; VC-6; SPC-4; FMF; HybRatio; khs.dsCH; khs.aaCH; khs.sssCH; khs.tsC; khs.sNH2; khs.dO; khs.ssO; khs.sF; LOBMIN; MDEC-12; MDEC-13; MDEC-22; MDEO-11; MDEO-12; MDEO-22; TopoPSA
	RF	MeanDecreaseAccuracy ^b^	MDEO-12; XlogP; khs.sssCH; ATSc5; FMF; MDEC-33; TopoPSA; MDEO-11; VC-5; MDEC-22
		MeanDecreaseGini ^b^	TopoPSA; MDEO-12; ATSc1; FPSA-2; khs.sssCH; nHBAcc; RNCG; XlogP; AMR; DPSA-2
	CT		9D: MDEO-12; Khs.sssCH; MDEO-11; VC-6; ALogp2; SCH-7; BCUTc-1h; C2SP2; BCUTp-1l
Antibiotic activity	SVM ^a^		38D: ALogP; BCUTw-1h; BCUTp-1l; DPSA-3; FPSA-3; FNSA-2; RPCG; RNCS; TPSA; Wnu1.unity; nAromBond; ATSc1; ATSc5; ATSm5; nBase; C2SP2; C3SP3; SCH-4; SCH-5; VCH-4; VCH-7; VPC-5; khs.sssCH; khs.tsC; khs.dssC; khs.ssO; khs.sF; nAtomLC; MDEC-13; MDEC-22; MDEC-24; MDEC-33; MDEO-11; MDEO-12; MDEO-22; MOMI-XZ; TopoPSA; XLogP
	RF	MeanDecreaseAccuracy ^b^	MDEO-12; MDEC-22; C2SP2; khs.dsCH; khs.sssCH; khs.dssC; VCH-5; MDEC-33; TopoPSA; XlogP
		MeanDecreaseGini ^b^	TopoPSA; ATSc1; FPSA-2; MDEO-12; RNCG; nHBAcc; MDEC-22; khs.sssCH; C2SP2; khs.dssC
	CT		16D: TopoPSA; C2SP2; VC-5; MDEC-22; XlogP; BCUTp-1h; VP-0; SCH-7; DPSA-1; Khs.dssC; Khs.ssCH2; Khs.sssCH; Khs.sssN; MDEO-12; THSA; VC-4

^a^ The selection of the descriptors was with the CFS (correlation-based feature subset selection) filter from Weka; ^b^ the mean decrease in accuracy and the mean decrease in Gini are two measures of importance for the descriptors using the RF algorithm.

The *MeanDecreaseAccuracy* parameter (Mean Decrease in Accuracy) of importance is considered more reliable than the *MeanDecreaseGini* parameter (Mean Decrease in Gini Coefficient) [44]. However, as was expected, there are several descriptors that are common to both measures for modeling the overall biological activity, antitumor and antibiotic activities (three, four and six out of ten, respectively) and the most important descriptor is the same in the overall activity model.

**Figure 2:**
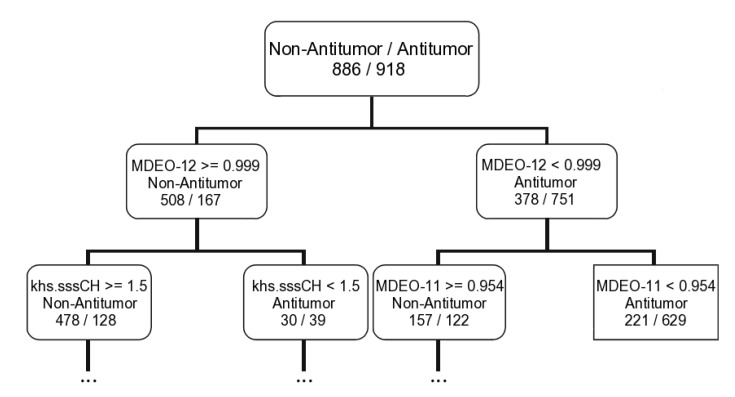
Representation of the first two rules of the antitumor classification tree derived with the CART algorithm for the training set.

Taking into account the *MeanDecreaseAccuracy* measure in the RF model for predicting the overall biological activity, antitumor and antibiotic activities it is possible to correlate those activities with the type of descriptors selected. For instance, the overall biological activity appears to be related with the electronic and molecular descriptors. The CPSA, BCUT and WHIM descriptors seem to have a main role in modeling the overall activity. The BCUT descriptors encode connectivity information and atomic properties of the molecule [45]. The WHIM descriptors are weighted holistic invariant molecular descriptors that are built in such a way to capture relevant molecular 3D information with respect to molecular size, shape, symmetry and atom distribution [46]. While the antitumor activity appears to be largely related with the topological descriptors. In addition, the molecular distance edge descriptors (MDE), which evaluate molecular distance edge descriptors for carbon, nitrogen and oxygen atoms, seems to be the most important type of the descriptors for modeling the antitumor activity (4 MDE descriptors out of the ten most important descriptors). Only the MDEO-12 (molecular distance edge between primary and secondary oxygen atoms) and the khs.sssCH (a fragment count descriptor that encode the presence of a tertiary carbon group in which it has three single bonds) descriptors were selected for all machine learning techniques (*i.e.*, CFS filter for SVM, the two measures of the RF, and CT). The MDEO-12 is the most important descriptors for the *MeanDecreaseAccuracy* parameter in the RF model and it is as well the first descriptor selected to build the antitumor classification tree (see a graphical representation of the first two rules of the antitumor tree in Figure 2).

**Scheme 5:**
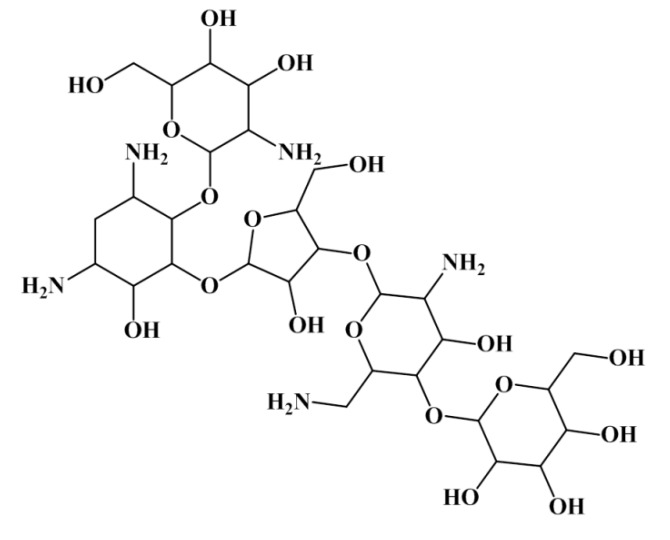
The mannosylparomomycin from the training set.

Using the first rule of the antitumor tree (MDEO-12 < 0.999 for antitumor compounds and MDEO-12 ≥ 0.999 for non-antitumor compounds) it was possible to correctly discriminate antitumor/non-antitumor 751/508 compounds, corresponding to ~70% of the training set and 372/245 compounds, corresponding to ~68% of the test set I. Training and test set I with 918/886 and 456/446 antitumor/non-antitumor compounds, respectively. The results were even impressive when only the “MDEO-12 < 0.999” rule was applied—from the 918 antitumor compounds 751 were correctly classified, ~82%, for the training set and 372 out of 456, ~82%, for the test set I. Only two of 16 lead-like antitumor MNPs and M_b_NPs that we had already proposed are classified as non-antitumor (IDs 567 and 580) by this rule. The remarkable performance of this descriptor in discriminating between antitumor and non-antitumor compounds has never been reported, however it was reported the importance of MDE descriptors in modeling chemical reactivity parameters [47], platelet-derived growth factor (PDGFR) inhibitors [48], Factor Xa inhibitors [48], cyclooxygenase-2 (COX-2) inhibitors [48], and dihydrofolate reductase (DHFR) inhibitors [49]. The MDEO-12 descriptor is known to codify the molecular size by taking into account oxygen atoms also characterizes polarity [48]. Inspection of the compounds belonging to the training set reveals that this descriptor provides an indication of the presence of oxygen-containing groups such glycosyl, amide, lactam, ester or lactone together with hydroxyl, carboxylic acid or ether functional groups. For example the aminoglycoside antibiotic, CID 215651, from the training set (see Scheme 5), has a high MDEO-12 descriptor value of 11.20.

**Figure 3:**
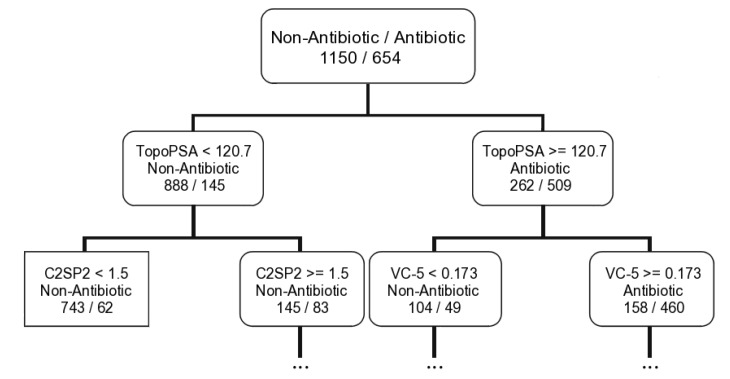
Representation of the first two rules of the antibiotic classification tree derived with the CART algorithm for the training set.

In turn, the antibiotic activity seems to be related mainly with the topological and constitutional descriptors. Six descriptors were selected for all machine learning techniques—one electronic (TopoPSA), two topological (MDEO-12 and MDEC-22—molecular distance edge between secondary carbon atoms), two constitutional (khs.sssCH, khs.dssC—a fragment count descriptor that codifies the presence of a quaternary carbon group in which it has two single bonds and one double bond), and one molecular (C2SP2—counts the number of sp2 secondary carbon atoms). The TopoPSA is the most important descriptor for the *MeanDecreaseGini* parameter in the RF model and it is as well the first descriptor selected to build the antibiotic tree (see a graphical representation of the antibiotic tree first rule in Figure 3).

Using the first rule of the antibiotic tree (TopoPSA ≥ 120.7 for antibiotic compounds and TopoPSA < 120.7 for non-antibiotic compounds) it was possible to correctly discriminate 509/888 and 267/416 antibiotic/non-antibiotic compounds for the training set (654/1150) and test set I (338/564), respectively. Subsequently the TopoPSA descriptor presents similar results for both antibiotic and non-antibiotic classes differently to the antitumor CT model where the MDEO-12 descriptor shows impressive discrimination ability only relatively to the antitumor compounds. Globally 1397 out of 1804 compounds, ~77%, were correctly classified by this rule with 509 out of 654 antibiotic compounds, 77%, and 888 out of 1150 non-antibiotic compounds, ~77%, correctly classified for the training set. Similar results were obtained for the test set I. Only three M_b_NP of 57 lead-like antibiotic MNPs and M_b_NPs that we had already proposed are classified as non-antibiotic (IDs 128, 483, 977) by this rule. In spite of the predictive power of the TopoPSA descriptor for modeling the antibiotic activity has never been reported, its importance in predicting P-glycoprotein [50] and cytochrome P450 [51] inhibitors was already noticed. Moreover, the high correlations of this descriptor with passive drug transport through membranes [43] and drug transport by multidrug resistance associated protein 1 (MRP1/ABCC1) [52] were also reported.

## 3. Material and Methods

### 3.1. Data Sets

The training and test set (I) were extracted from the PubChem [53,54] database, searching by type of biological activity (e.g., antitumor, antibiotic, antimicrobial, antifungal, antimalarial, anti-HIV) and their chemical structures saved in the SMILES data format. The training and test sets comprise of the following biological categories of compounds: 1367 antitumor, 992 antibiotic, 59 antifungal, 11 antimalarial, one anti-HIV, 79 anti-microbial, 152 cytotoxic and 206 non-active. It is important to refer that some compounds have more than one bioactivity record. The non-active compounds were selected on the basis that were at least screened for one biological activity. The whole data set (2706 compounds) was divided into a training set of 1804 compounds and a test set of 902 compounds, which was used for the development and external validation of the QSAR classification models. The approximate 2:1 partition was carried out randomly according to the antitumor, antibiotic, and non-active categories in such a way that both sets span the biological diversity of the data set. A further test set (test set II), which comprises 1192 MNPs and M_b_NPs was extracted from the AntiMarin database [3,14,15]. The chemical structures of the MNPs were downloaded in the MDL SDF format. As some drug molecules from PubChem originated from natural sources origin, there is some overlapping between the two databases. After assembling these two databases, the duplicates were removed, although the chirality was taken into account, racemic compounds (or cases where the stereochemistry was not indicated) were considered as one of the possible stereoisomers. The SMILES strings of the three different data sets, the natural source for the compounds of test set II, and the corresponding experimental and predicted activities are available as Supplementary Information.

### 3.2. Molecular Descriptors

The molecular structures were standardized by normalizing tautomeric groups and by removing small disconnected fragments using JChem Standardizer tool version 5.12.3 (ChemAxon Ltd., Budapest, Hungary). Three-dimensional models of the molecular structures were generated with CORINA version 2.4 (Molecular Networks GmbH, Erlangen, Germany). Empirical Molecular descriptors were then calculated using the CDK Descriptor Calculator 1.3.2 [55,56]. A total of 270 descriptors were calculated including electronic, topological, geometrical, constitutional and hybrid (BCUT and WHIM) descriptors.

### 3.3. Selection of Descriptors and Optimization of QSAR Classification Methods

After the removal of constant descriptors, multilinear regressions (MLR) were built with Weka 3.6.5 [57,58,59] to select descriptors by the M5 method, using the training set. In accordance with this preliminary work, 232 descriptors were selected from CDK collection. In the quest for QSAR classification models with the minimum possible number of descriptors, feature selection was further performed with the CFS (Correlation-based Feature Subset Selection) algorithm [60] implemented in Weka 3.6.5. This heuristic takes into account the usefulness of individual descriptors for predicting the activitiy (*i.e.*, overall biological, antitumor, or antibiotic) together with the level of intercorrelation among them. The experiments for comparing the descriptors for each one of three models were conducted with the AttributeSelectedClassifier routine of Weka with the CfsSubsetEval option as descriptor evaluator and the BestFirst or GreedyStepwise option as search method. This procedure selects descriptors with the CFS algorithm within a ten-fold cross-validation procedure and *k* nearest neighbor (KNN) algorithm as ML technique.

### 3.4. ML Techniques

The KNN algorithm [61] predicts the activity for a compound by majority voting of the *k* most similar compounds in the training set. For example, for *k* = 3, if two of the three most similar compounds relatively to the query compound are active the query molecule will be classified as active. It was applied here with the Weka 3.6.5 software using a *k* of 10 (10 most similar neighbors of the query compound), Euclidean distances (as measure of similarity), and contributions of neighbors weighted by the inverse of distance.

CTs were grown using R program, version 2.13.1, using the RPART library, which implements the CART algorithm, with the default parameters [62,63]. A classification tree is sequentially constructed by partitioning compounds from a parent node into two child nodes. Each node is produced by a logical rule, usually defined for a single descriptor, where objects below a certain descriptor’s value fall into one of the two child nodes, and objects above fall into the other child node. The prediction for a compound reaching a given terminal node is obtained by a majority vote of the objects (in the training set) reaching the same terminal node. The CT models were built using the 232 CDK descriptors.

A RF [64] is an ensemble of unpruned classification trees created by using bootstrap samples of the training data. The best split at each node was defined among a randomly selected subset of descriptors. Prediction is made by a majority vote of the classification trees in the forest. Additionally, performance is internally assessed with the prediction error for the objects left outin the bootstrap procedure (internal cross-validation or OOB estimation). The method quantifies the importance of a descriptor by the increase in misclassification occurring when the values of the descriptor are randomly permuted, correlated with the mean decrease in accuracy parameter, or by the decrease in a node’s impurity every time the descriptor is used for splitting, correlated with the mean decrease in the Gini coefficient parameter. RFs also assign a probability to every prediction on the basis of the number of votes obtained by the predicted class. A measure of similarity between two objects can be calculated from the number of trees in the ensemble that classify the two objects in the same terminal node. RFs were grown with the R program, version 2.13.1, using the Random Forest library [63]. The RF models were built with 500 trees and using the set of 232 CDK descriptors. As a result of the nature of two-class imbalance in the overall activity model, this problem was alleviated setting the class weights to 50:50.

SVM map the data into a hyperspace through a nonlinear mapping (a boundary or hyperplane) and then separate the two classes of compounds in this space. The boundary is positioned using examples in the training set that are known as the support vectors. With nonlinear data, kernel functions can be used to transform it into a hyperspace where the classes become linearly separable. In this study, SVM were established with the Weka 3.6.5 program, using the LIBSVM package [65,66,67,68]. The type of SVM was set to C-SVM-classification and the kernel function was the radial basis function. The parameter C of the C-SVM-classification was optimized in the range of 10–500 and the default γ parameter in the kernel function was used. The descriptors selected by the CFS procedure were normalized and used to develop the classification models. The active and non-active classes of the overall activity model were set to the weights of 10:90, respectively.

## 4. Conclusions

The results suggest that the implemented computer-aided approach could be used to predict the bioactivity of new, or existing NPs without bioactivity records, and by this way identify and propose lead compounds *en route* to a specified activity. The result of the application of this approach is the reduction in great extent the number of compounds used in real screens.

The obtained results for the presented screening of possible lead compounds *en route* to antitumor and antibiotic drugs were also externally supported by the publication in the literature as active compounds of some of the compounds proposed and initially classified in the AntiMarin database as non-active compounds or without activity record. Higher levels of accuracy will be expected if larger data sets of molecules are used to train the models. The use of other levels of descriptors, to better encode the main aspects found to be relevant for classification in the presented work, could also be useful to improve the classification ability of the models. This will be an interesting approach in future work.

## Supplementary Files

Supplementary File 1 Supplementary Information (XLSX, 1535 KB)
